# Mental health professionals’ perceived barriers and enablers to shared decision-making in risk assessment and risk management: a qualitative systematic review

**DOI:** 10.1186/s12888-021-03304-0

**Published:** 2021-11-25

**Authors:** Nafiso Ahmed, Sally Barlow, Lisa Reynolds, Nicholas Drey, Fareha Begum, Elizabeth Tuudah, Alan Simpson

**Affiliations:** 1 Centre for Mental Health Research, School of Health Sciences, City University of London, Northampton Square, London, EC1V 0HB UK; 2 School of Nursing, Midwifery and Allied Health, Buckinghamshire New University, 106 Oxford Rd, Uxbridge, UB8 1NA UK; 3 Centre for Health Services Research, School of Health Sciences, City University of London, Northampton Square, London, EC1V 0HB UK; 4 Health Service and Population Research, David Goldberg Centre, Institute of Psychiatry, Psychology & Neuroscience, King’s College London, De Crespigny Park, London, SE5 8AF UK; 5 South London and Maudsley NHS Foundation Trust, Maudsley Hospital, 111 Denmark Hill, London, SE5 8AZ UK; 6 Florence Nightingale Faculty of Nursing, Midwifery & Palliative Care, James Clerk Maxwell Building, King’s College London, 57 Waterloo Road, London, SE1 8WA UK

**Keywords:** Decision making, Mental health, Risk assessment, Risk management, Systematic review

## Abstract

**Background:**

Risk assessment and risk management are fundamental processes in the delivery of safe and effective mental health care, yet studies have shown that service users are often not directly involved or are unaware that an assessment has taken place. Shared decision-making in mental health systems is supported by research and advocated in policy. This systematic review (PROSPERO: CRD42016050457) aimed to explore the perceived barriers and enablers to implementing shared decision-making in risk assessment and risk management from mental health professionals’ perspectives.

**Methods:**

PRISMA guidelines were followed in the conduct and reporting of this review. Medline, CINAHL, EMBASE, PsycINFO, AMED and Internurse were systematically searched from inception to December 2019. Data were mapped directly into the Theoretical Domains Framework (TDF), a psychological framework that includes 14 domains relevant to behaviour change. Thematic synthesis was used to identify potential barriers and enablers within each domain. Data were then matched to the three components of the COM-B model: Capability, Opportunity, and Motivation.

**Results:**

Twenty studies met the eligibility criteria. The findings of this review indicate that shared decision-making is not a concept commonly used in mental health services when exploring processes of risk assessment and risk management. The key barriers identified were ‘power and best interest’ (social influences) and ‘my professional role and responsibility’ (social/professional role and identity). Key enablers were ‘therapeutic relationship’ (social influences) and ‘value collaboration’ (reinforcement). The salient barriers, enablers and linked TDF domains matched COM-B components ‘opportunity’ and ‘motivation’.

**Conclusion:**

The review highlights the need for further empirical research to better understand current practice and mental health professionals’ experiences and attitudes towards shared decision-making in risk assessment and risk management.

**Supplementary Information:**

The online version contains supplementary material available at 10.1186/s12888-021-03304-0.

## Background

In mental health services, Shared Decision Making (SDM) is a means of delivering recovery orientated care through involving individuals in decisions about their care. For a decision to be ‘shared’ it must involve: at least two participants, the sharing of information, and a decision that is made and agreed upon by all parties [1]. These criteria are reflected in a shared decision model [2], which proposes that SDM occurs when all participants are informed, involved, and influential in the decision-making process. It is, however, emphasised that the three SDM components are on a sliding scale of influence that is dependent on context, capacity and desire to influence [2].

In shared decision-making, the aim is to recognise and utilise the unique expertise of healthcare professionals and services users to produce better decisions, and potentially better outcomes. While healthcare professionals may be experts in diagnosis, aetiology, prognosis, treatment options, and outcome probabilities [3]; service users are experts about the impact of the condition on their lives, their preferences, their personal attitudes towards risks, and often know what works best for them regarding their condition and treatment [4].

Studies report positive effects of SDM interventions on patient outcomes within different mental health populations. A randomised control trial (RCT) for people with depression reported a positive impact on patient participation in treatment decision-making and patient satisfaction [5]. Another RCT of an intervention for people with schizophrenia found SDM improved social recovery [6]. A pilot trial of a SDM intervention with veterans with post-traumatic stress disorder (PTSD) found positive impacts on patients’ receptivity to evidence-based treatment [7]. In contrast, some studies report no significant effect of SDM on clinical outcomes for people with severe mental illness [8] and depression [9], although they acknowledge that further long-term work may be needed to detect an effect.

Shared decision-making is endorsed and advocated in international healthcare policy [10, 11]. Research has found that both service users and professionals support SDM. A qualitative research synthesis examining stakeholders’ attitudes towards SDM in mental health reported that service users valued their voice being heard, listened to, and supported to express themselves in encounters with professionals [12]. Several barriers to SDM were identified from the service user’s perspective, including feelings of perceived inadequacy, fear of being judged and a lack of trust. Barriers to SDM for professionals included: the service user lacking cognitive capacity or insight; where stigma negatively influenced the service user’s attitude towards SDM; and the professional’s own attitudes, motivation, willingness, empathy, and ability to engage and implement SDM. Professionals also highlighted challenges surrounding the competing priorities of their role, mainly them being accountable and responsible for managing risk.

Implementing SDM may pose challenges when there are concerns about the potential risks to self or others [13, 14]. In these circumstances, mental health professionals (MHP) may not feel able to engage service users in decisions about their care. Potential barriers cited in the literature include inadequate training in suicide prevention [15]; fears about negative adverse reaction from individuals who pose a risk to other [16] and the ‘blame culture’ observed in mental health care [17], whereby MHPs are increasingly fearful of culpability and litigation. It has been suggested that this has resulted in more defensive or risk-averse practice intended to prevent harm [18, 19].

Risk in mental health care is often used to refer to the possibility of an adverse event, outcome or behaviour arising from the unwanted actions of the service user [20, 21]: notably risk of harm to self, others, or both, and may include self-harm, suicide, or violence. Risk also signifies the vulnerabilities that a person with mental illness may be exposed to, such as side effects from medication, exploitation, victimisation, bullying, and discrimination [22, 23]. These risks occur frequently but are considered less in the assessment and management of risks [24].

Risk Assessment (RA) and Risk Management (RM) are the mechanisms used by MHPs to identify and minimise risk. There are three main approaches to assessing risk in mental health care: unstructured clinical judgement, actuarial methods and structured clinical judgement [25]. Unstructured clinical judgement typically involves professionals making judgements based on their clinical experience, opinion, intuition or ‘gut feeling’. Actuarial methods provide the assessor with a statistical means to combine information and calculate risk [26]. The subjective nature and poor predictive accuracy of these approaches have resulted in recommendations for them not to be used on their own in clinical practice [27]. Structured clinical judgement is considered the best approach to assessing risk [28]; this involves the use of a standardised RA tool to aid a professional in their clinical judgement [25].

Nonetheless, studies have found wide variability in the methods used to assess risk in UK mental health services [29] and forensic services in Australia and New Zealand [30]. These studies agree that a more consistent approach to RA is needed in mental health services. A multitude of evidence-based guidance is available to help standardise the process and support professionals in their assessment of risk [28, 31–33]. A model for assessing suicidality, for example, provides guidance on the importance of language, the structure of the clinical interview, questioning, actuarial tools and risk categorisation [31].

Risk management is informed by the RA and includes the key actions or strategies that are designed to prevent or limit undesirable outcomes. Strategies may include treatment, supervision (i.e. help with planning daily activities), or monitoring (i.e. identifying and looking out for early warning signs) [28]. Several RM and safety planning interventions have been developed that can be used to mitigate, contain or improve RM [34–36].

The need to involve service users in the RA and RM process has been advocated in current professional guidance, policy, and research [28, 33, 37]. Involving service users is a means of minimising the gap between professionals and service users’ perspectives of risk [38, 39] and thus, ensuring that the plan developed meets the individual’s needs [33]. This can lead to more accurate prediction and management of risk. Another potential benefit of involvement is that the individual is empowered to take responsibility for their choices, which can be a motivator for change [40]. It has been suggested that service user involvement can improve confidence and self-management skills, which may have long term impacts on reducing dependency on services, thereby increasing cost-effectiveness [37].

The UK Department of Health (DH) best practice guideline, specifically recommends SDM. Studies have shown, however, that service users are often unaware that a RA has taken place [41, 42].

Although Higgins, Doyle [24] found that more than three-quarters of MHPs reported ‘always’ involving service users in risk assessment (77.8%) and safety planning (78.4%), only 50% of the respondents reported that they ‘always’ informed service users about their risk level, while only 43% of the respondents reported that they ‘always’ developed a shared responsibility with the service user for safety. Despite professionals reporting a high rate of service user involvement, these findings suggest that SDM is not routinely nor fully implemented.

A recent systematic review of mixed methods studies explored the service users’ perspective of helpful RM practices within mental health services [43]. Two categories of beneficial RM practices were identified: interpersonal relationships and communication; and agency and autonomy. A key finding was that trust fosters openness in relationships and enables discussion of risks, especially when service users felt that their distress was understood or their accounts were validated by professionals. Service users preferred professionals to maintain responsibility for RM initially but that eventually (at their own pace) they wished to regain control.

Other systematic reviews in this field have focused on interventions that promote SDM in RA and RM in forensic mental health settings [36, 44]. A qualitative synthesis of research examining professionals attitudes towards SDM in the broader field of mental health exists [12], however, the authors acknowledge that the rigour of a full systematic review was not adopted. There is currently no systematic review of MHPs’ experiences and attitudes towards implementing SDM in the assessment and management of risk. A synthesis of studies will improve our understanding of the discrepancies in reported practice and identify factors that may help or hinder its implementation. The specific review question was:

What do mental health professionals perceive as the barriers and enablers to SDM in RA and RM?

## Methods

This review was conducted in line with the Preferred Reporting Items for Systematic Reviews and Meta-Analyses (PRISMA) guidelines [45]. The protocol is registered on PROSPERO (CRD42016050457).

### Eligibility criteria

The SPIDER framework (sample, phenomenon of interest, design, evaluation, research type) was used to specify eligibility criteria [46]. An additional S was added to capture the ‘setting’ criterion of adult mental health services. The SPIDER framework is a tool for developing a search strategy that has been designed from the PICO tool, specifically for reviews that aim to synthesise qualitative and mixed-method research studies. Due to limited resources, only studies written in English were included in the review. Table 1 lists the inclusion and exclusion criteria.

**Table 1:** Inclusion and exclusion criteria based on SSPIDER

	Inclusion	Exclusion
Sample/ Population	Studies that included mental health professional (MHP) participants: • Any member of staff responsible for risk assessment and risk management (i.e. mental health nurse, social worker, psychologist, occupational therapist and doctor/psychiatrist). • Mixed population (e.g. service users and MHP) studies were only included if the results were reported separately and data easily extractable.	• Studies that did not include MHPs (e.g. participants are all service users) • Studies conducted on students, trainees, peer support workers or those who are not responsible for risk assessment and risk management.
Setting	Adult mental health services (both inpatient and outpatient services) in any geographical location	• Non-mental health related studies (i.e. physical health or learning disability). • Studies set in older adult, child and adolescent mental health services (CAMHS) or drugs/alcohol services.
Phenomenon of Interest	Studies that reported on MHPs’ experiences and attitudes towards Shared Decision Making (SDM) in risk assessment (RA) and risk management (RM) with people with mental health problems. Studies that provided possible barriers and enablers to SDM in RA and RM as perceived by MHPs. For the purpose of this review: • For a decision to be a ‘shared’ decision it must include at least two participants (i.e. professional and service user), the sharing of information and a decision that is made and agreed upon by all parties • Based on Stacey et al’s (2015) ‘Three I’s Scale of Influence’ model, SDM requires all participants to be informed, involved and influential. Therefore, studies that discussed ‘working in collaboration’ or ‘service user involvement’ were included • Risk assessment may include statistical/actuarial tools, traditional clinical judgement or structured clinical judgement (combined)	
Design of study	All study designs that produced original qualitative data, or mixed-methods studies that included a qualitative component	Studies that reported primarily quantitative data or where no qualitative analysis had been undertaken.
Evaluation	Qualitative outcome methods that measured MHPs’: experiences of; attitudes towards; or perceived barriers and enablers to SDM in RA and RM	
Research type	Original empirical studies. No restriction on publication status.	• Systematic reviews • Editorials • Opinion pieces • Letters and similar materials
Language	Only studies written in English.	

### Search strategy

The EBSCOhost and Ovid Online platforms were used to search six electronic bibliographic databases: MEDLINE; EMBASE; PsycINFO; CINAHL; AMED and Internurse. Databases were searched from inception. The last search was completed on the 4th December 2019.

The search strategy used a combination of medical subject headings (MeSH) and free text key terms related to concepts of ‘mental health’, ‘health professionals’, ‘experiences’, ‘shared decision making’, ‘risk assessment’ and ‘risk management’. A full electronic search strategy is presented in Additional file 1.

Two grey literature databases were also searched for relevant unpublished empirical research studies; Bielefeld Academic Search Engine (BASE) and Open Grey. Citation chaining was performed on all articles selected for inclusion to identify further studies of interest, and this involved searching the reference lists (backward chaining) and using Google Scholar to identify and review papers that had cited the included articles (forward chaining).

### Study selection

Search results were imported into a systematic review management software EPPI-reviewer 4 [47] and duplicates removed. Two-stage screening was undertaken: stage 1 screened the titles and abstracts of studies against the eligibility criteria; stage 2, further assessed full-text of potential studies against the eligibility criteria. Study authors were contacted if more information was needed.

To minimise risk of bias, two authors (NA and FB) independently assessed titles and abstracts, and subsequently, full-text articles. A full-text review was carried out if at least one of the reviewers believed that the study met the inclusion criteria at the title and abstract screening stage. At full-text review, any discrepancies regarding eligibility were resolved by consensus and in consultation with a third author (AS/LR). Also, studies were included only once if they had multiple articles. The original or most relevant to the review question was used as the primary article for the study’s results.

The ‘Three I’s Scale of Influence Model’ [2] was used as a framework for study selection. Studies that reported on a least one of the three components (informed, involved and influential) of SDM in RA and RM were included. Stacey, Felton [2] definitions of the SDM components can be found in Additional file 2.

### Data extraction

An electronic data extraction form was devised and piloted on two of the included studies. The following data items were extracted: author(s), publication year, research question/aim, geographical location, sample size, setting, data collection, and method of analysis. The entire results sections, including direct quotations and author interpretations were imported directly into NVivo 11 software [48]. For studies with multiple publications, results were extracted and collated from all the linked reports but only one publication was used as the source of study results. Data extraction was carried out by the first author (NA) and cross-checked by a second author (SB): disagreements were resolved through discussion.

### Quality appraisal

Dixon-Woods, Shaw [49] prompts were used to assess the quality and relevance of individual studies within this review. These prompts focus on the universal features of qualitative research and have been devised to *‘sensitise appraisers to the various dimensions of articles that require evaluation’* (p224). Two reviewers (NA and AJ or UF – see acknowledgements) read the papers independently and answered a series of questions on the quality appraisal checklist (e.g., Are the research questions clear?). They recorded their response as Yes (Y), No (N), Can’t tell (−). A rating system was then used to categorise the papers: Key paper (meets all quality criteria and clearly fits with review question); Satisfactory (meets most quality criteria and fits well to review question); Unsure (mixed responses to quality criteria and lack of clarity regarding relevance to review question); and Poor (does not meet quality criteria) [50]. No studies were excluded based on methodological quality; however, a sensitivity analysis (described below) was conducted to see the impact of removing lower-rated studies on the review findings. Any disagreements were discussed in full, and a rating was agreed (Additional file 3).

### Data synthesis

The Theoretical Domains Framework (TDF) was used to explore the factors that influence the implementation of SDM in RA and RM with individuals with mental illness. The TDF is a behaviour change framework developed by a group of experts to simplify and integrate the large number of psychological theories relevant to behaviour change [51]. The TDF has been used by researchers across a range of healthcare settings to identify determinants of behaviour, namely the barriers and enablers to implementation, and to inform intervention design [52]. The original TDF has 12 domains derived from 33 health and social psychology theories and 128 key theoretical constructs. The framework was later validated and refined by Cane, O’Connor [52] to include 14 theoretical domains. The revised version of the framework was used in this review, Cane et al. (2012) definition of each domain is presented in (Additional file 2).

The Capability, Opportunity, and Motivation (COM-B) model was then used to condense the relevant TDF domains into three components that interact to predict behaviour. The model was developed as part of the broader framework of the behaviour change wheel [53] and provides a basis for intervention design. Each component of the COM-B model is divided into sub-components that capture important distinctions. Capability can be physical (e.g. skills) or psychological (e.g. interpersonal skills and knowledge) and represents an individual’s capacity to carry out the behaviour. Opportunity can be physical (e.g. environmental factors) and social (e.g. social influences) and is defined as all the factors that lie outside the individual that influence the behaviour. Motivation can be reflective (e.g. beliefs, intentions) or automatic (e.g. emotions) and characterises the brain processes that drive behaviour [53]. The most relevant TDF domains and linked components that are likely important to changing behaviour were identified [52].

The data synthesis process drew on established analysis methods recommended in the TDF guidelines [54], and used in previous studies applying the TDF [55–57]. Data synthesis involved the following six stages:

#### Step 1: developing a coding manual

A coding guide was developed based on the definitions of the three components of SDM [2], and the 14 domains and 84 constructs from Cane, O’Connor [52]. To provide guidance and confidence that a piece of text represents a domain, statements of how the domain applies to the research context were also included in the coding guide.

#### Step 2: pilot coding exercise

To ensure consistency between coders and refine the coding guideline, two coders (NA and ET) jointly coded the extracted findings from two randomly selected included papers. Any disagreements were discussed until consensus was reached; where consensus could not be reached a third researcher was consulted. The final version of the coding guide is included in Additional file 2.

#### Step 3: coding papers and assessing reliability

Two researchers (NA and ET) independently coded the extracted findings from the remaining included papers using the coding guideline and via NVivo 11 software [48]. Findings relating to the target behaviour were coded to the SDM components [2], whereas potential barriers and enablers identified within the included papers were coded to the 14 domains of the TDF [52]. For example, the statement *‘“[the risk assessment is] one thing … you never discuss with service users just in case it alarms them”’* was coded to the ‘informed’ component and the ‘beliefs about consequences’ domain. If the participant’s response or the author’s interpretation represented more than one TDF domain, the text was coded to multiple domains. For example, *“You know that you’re going to have suicide risk but you think well, the psychologists will deal with that bit … so to want to deal with it, even as part of the overall care, I think you’d want some type of supervision”* was coded to both “social professionals’ role and identity” and “social influences”.

Inter-coder reliability was assessed by calculating the percentage agreement/disagreement (prior to consensus being reached), to measure consistency in coding within and across domains [58]. Reliability between two coders is considered acceptable if percentage agreement > 60% is achieved [54]. Discrepancies in coding were addressed by NA and ET with a consensus reached by discussion. AS was available to resolve any disputes over discrepancies; however, this was not required.

#### Step 4: developing overarching themes

Data within the domains were further analysed by the lead researcher (NA) using thematic synthesis [59]. Text coded into each domain were compared across papers, and findings representing similar ideas were grouped together. An overarching theme was then generated to categorise the initial themes. The overarching themes represent the specific factor perceived to influence SDM in RA and RM. For example, findings that suggest rapport, alliance or connection facilitate discussion about risk with service users were categorised as ‘therapeutic relationship’.

#### Step 5: mapping the COM-B model to the TDF domains

The relevant TDF domains were matched to the COM-B components [53]. The lead researcher (NA) drew on the links between the TDF domains and COM-B components identified by a group of experts in a consensus exercise reported in Cane, O’Connor [52]. The most relevant TDF domains (and themes within) were identified based on a frequency count of studies by domain. The TDF domains (and themes within) identified in at least 60% (*n* = 11) of the included studies were considered salient in understanding the target behaviour.

#### Step 6: sensitivity analysis

A sensitivity analysis was carried out to determine whether the methodological quality of studies impacted on the findings of the review. The results from the lowest-rated studies were removed from the synthesis to see if this influenced the key themes originally identified. No studies were excluded based on methodological quality.

## Results

### Study selection

**Fig. 1:**
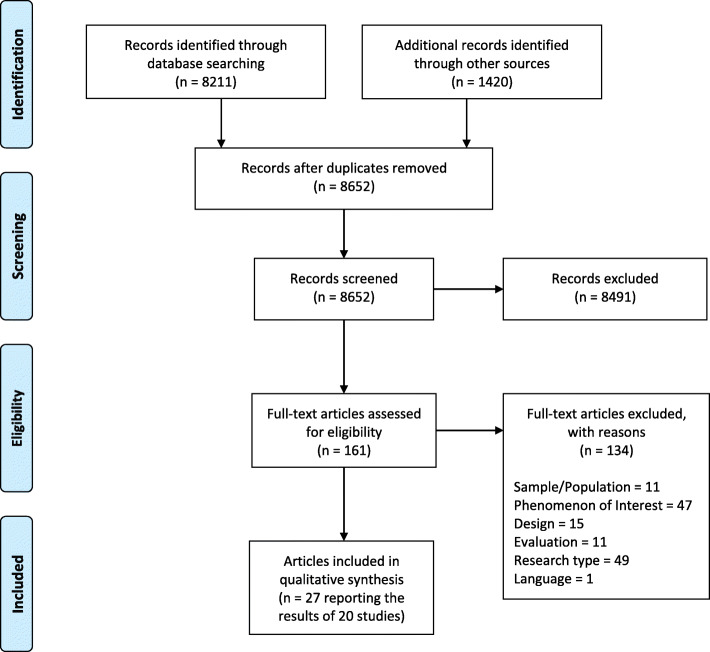
A PRISMA flow diagram detailing the search strategy and results (Moher et al., 2009) [45]

A total of 8211 papers were yielded in the databases searches; and 1420 additional papers were included from other sources. After the removal of duplicates, a total of 8652 papers were eligible for screening. Following title and abstract screening, 8491 papers were excluded, and 161 full text papers were reviewed; 134 papers were excluded at full-text, and 20 studies (reported in 27 papers) met the inclusion criteria for this review. The PRISMA diagram of study selection can be seen in Fig. 1.

### Quality appraisal

All papers gained the rating of either key paper (*n* = 9) or satisfactory (*n* = 11). Papers were rated satisfactory if they did not meet all of the quality criteria and/or did not clearly fit with the review question. For example, papers that reported on specific risk decisions i.e. decision-making regarding neuroleptic medication [60]; specific RM practices i.e. clinician-patient alliance during mechanical restraint [61]; or contained very limited findings relevant to the review question [62] were rated satisfactory. Quality appraisal of the included studies can be seen in Additional file 3.

### Study characteristics

Over half of the included studies were conducted in the UK (*n* = 11), two in Belgium and the remaining studies in Australia, Canada, Taiwan, Denmark, Sweden, Italy, and Norway. The papers were published between 1999 and 2019 and were predominantly qualitative in design (*n* = 18). Semi-structured interviews were the most common data collection method (*n* = 15); four studies utilised focus groups [61, 63–65]; and one used in-depth interviews [60]. Three studies used unstructured observation in addition to semi-structured interviews [66–68]. One study surveyed participants before conducting the qualitative interviews [69], and one described using a mixed-methods approach [65] comprising of focus groups and a quantitative analysis technique (i.e., inductive content analysis). Their findings, however, included several illustrative quotes that were deemed relevant to the review question.

Over half of the studies gathered data from adult psychiatric/forensic inpatient settings. (*n* = 12). Other settings included adult community mental health teams (*n* = 4) or both inpatient and community mental health settings (*n* = 4).

The included studies focused on a range of risk issues including suicidality (*n* = 7); risk to others [16]; self-neglect [70] and violence [64]. Two of the studies explored safety and risk within the broader topic of care-planning [20, 62]. Other studies explored specific RM practices [61, 65, 68, 71]; the tension between promoting recovery and managing risk [66, 72]; and risk-minimisation and risk-taking [73]. One study examined clinicians’ perspectives of supporting service users who wished to discontinue from medication, which is a form of risk-taking [60]. Several of the included studies [16, 20, 66, 71, 74] had multiple publications from the same study [42, 75–80]. The characteristics of the included studies are summarised in Table 2.

**Table 2:** Characteristics of included studies

Author (Year)	Research aim	Location	Population	Data collection method	Data analysis method	Quality rating
Coffey et al. (2017) [20] *Linked papers* (Simpson et al., 2016a [75], Simpson et al., 2016b [76])	To examine what patients, family members and workers say about risk assessment and management.	UK	*N* = **67** Community mental health teams Senior managers (*N* = 12); Senior practitioners (*N* = 27); Care coordinators (*N* = 28)	Semi-structured interviews	Thematic analysis	Key Paper
Gunstone (2003) [70]	To explore the experiences and perceptions of community mental health workers in assessing and managing the risk of self-neglect or severe self-neglect in people with serious mental health problems.	UK	*N* = **7** Community mental health team and assertive outreach team Community mental health workers (*N* = 7)	Semi-structured interviews	Thematic content analysis	Satisfactory
Holley et al. (2016) [72]	To explore how risk management practice impacts upon the implementation of recovery- oriented care within community mental health services.	UK	*N* = **8** Community mental health teams Social worker (*N* = 3); Occupational therapist (*N* = 1); Nurse (*N* = 2); Psychiatrist (*N* = 2)	Semi-structured interviews using vignettes	Grounded theory	Key Paper
Langan (2008) [16] *Linked publication* (Langan and Lindow, 2004 [42])	To explore how MHPs assessed risk to others and the extent to which they involved service users. To ascertain service users’ knowledge of, and involvement in, risk assessment.	UK	*N* = **46** Adult psychiatric inpatient setting Psychiatrist (*N* = 14); Nursing (*N* = 22); Social worker (*N* = 5); Psychologist (*N* = 2); Occupational therapist (*N* = 1); Unqualified (*N* = 2)	Semi-structured interviews	Thematic analysis	Key Paper
Woods (2013) [63]	To identify and describe the nature and extent of current risk assessment and management approaches used in the adult inpatient mental health and forensic units	Canada	*N* = **48** Adult inpatient mental health and forensic units Psychiatric Nurse (*N* = 33); Registered Nurse (*N* = 2); Licensed Practical Nurse (*N* = 1); Special Care Aide (*N* = 7); Social Worker (*N* = 2); Student Nurse (*N* = 1); Other (*N* = 2)	Focus groups	Thematic analysis	Satisfactory
Barnicot et al. (2017) [71] *Linked paper* (Insua-Summerhays et al., 2018 [77])	To understand how staff and patients experience negotiating the balance between privacy and safety during decision-making about continuous observation.	UK	*N* = **31** Adult psychiatric inpatient setting Nursing (*N* = 9) Unqualified nursing staff (*N* = 12); Clinical team leader (*N* = 2); Ward manager (*N* = 3); Modern matron (*N* = 1); Consultant psychiatrist (*N* = 3); Consultant clinical psychologist (*N* = 1)	Semi-structured interviews	Thematic analysis	Key Paper
Felton et al. (2018) [66] *Linked paper* (Felton et al., 2018) [78]	To examine MHPs’ experiences of potential contradictions between promoting recovery and managing risk in decision-making.	UK	*N* = **17** Acute inpatient ward and assertive outreach team Mental health nurse (*N* = 4); Ward charge nurse (*N* = 1); Consultant psychiatrist (*N* = 3); Community mental health nurse (*N* = 7); Community support worker (*N* = 1); Support worker team manager (*N* = 1)	Unstructured observations and semi-structured interviews	Case study theory building approach	Key Paper
Awenat et al. (2017) [81]	To investigate the experiences and perceptions of staff working with in-patients who are suicidal	UK	*N* = **20** Adult psychiatric inpatient setting Qualified nurse (*n* = 8); Nursing assistant/support worker (*N* = 2); Psychiatry (*N* = 4); Allied health professional (*N* = 6)	Semi-structured interviews	Thematic analysis	Key Paper
Sun et al. (2006) [67]	To explore and examine psychiatric nurses’ and patients’ perceptions of the care offered to patients with suicidal ideations on psychiatric wards	Taiwan	*N* = **15** Acute psychiatric ward and psychiatric stress ward Registered Nurses (*N* = 15)	Participant observation and semi-structured interviews	Grounded theory	Satisfactory
Forsberg et al. (2018) [60]	To examine the processes involved in clinicians’ decision-making, specific to neuroleptic discontinuation.	UK	*N* = **12** Adult community mental health team, early intervention service or recovery team Psychiatrist (*N* = 5); Mental Health Nurse (*N* = 7)	In-depth interviews	Grounded theory	Satisfactory
Vandewalle et al. (2019a) [82]	To uncover and understand the core elements of how nurses on psychiatric wards make contact with patients experiencing suicidal ideation.	Belgium	*N* = **19** Adult psychiatric wards Nurses (*N* = 19)	Semi-structured interviews	Grounded theory	Key paper
Nielsen et al. (2018) [61]	To report on forensic mental health clinicians’ experiences of the clinician-patient alliance during mechanical restraint.	Denmark	*N* = **17** Forensic mental health setting: secure unit and rehabilitation unit Nurse Assistant (*N* = 1) Social and Healthcare Assistant (*N* = 8) Nurse (*N* = 8)	Focus groups	Thematic analysis	Satisfactory
Nyman et al. (2020) [64]	To explore mental health nurses’ experiences of risk assessments within their care planning and management of risks for violence by forensic patients.	Sweden	N = **15** Forensic psychiatric Wards Mental Health Nurse (*N* = 15)	Focus groups	Content analysis	Satisfactory
Rimondini et al. (2019) [65]	To investigate the critical issues and strategies related to psychiatric patients’ empowerment in risk management.	Italy	*N* = **95** Various mental health settings Psychiatric nurse (*N* = 67); Healthcare and Social Assistance Operator (*N* = 10); other mental health professional, e.g., Psychiatrists, clinical psychologists, (*N* = 18).	Focus groups	Content analysis	Key paper
Vandewalle et al. (2019b) [83]	To uncover and understand the actions and aims of nurses in psychiatric hospitals during their interactions with patients experiencing suicidal ideation.	Belgium	*N* = **26** Adult psychiatric wards Nurse (*N* = 26)	Semi-structured interviews	Grounded theory and constant comparison analyses	Key paper
Coffey et al. (2019) [62]	To explore participants’ views and experiences of care planning and co-ordination, safety and risk, recovery and personalisation, and the context within which these operated.	UK	*N* = **31** Acute inpatient ward Nurses, ward managers, occupational therapists, psychologists and psychiatrists (*N* = 31)	Semi-structured interviews	Framework method	Satisfactory
Lees et al. (2014) [69]	To explore the experiences and needs that mental health care consumers had of suicidal crisis, the degree to which those needs were met, the role that mental health nurse engagement played in that context, and the key factors suggested to impact on the quality of care.	Australia	*N* = **11** Adult inpatient and community settings Mental Health Nurse (*N* = 11)	Semi-structured interviews	Critical discourse, constant comparative and content analysis	Satisfactory
Hagen et al. (2017) [74] *Linked papers* (Hagen et al., 2017a [79], Hagen et al., 2017b [80])	To explore and compare therapists’ and mental health nurses’ experiences of caring for suicidal inpatients in light of ethics of care and ethics of justice.	Norway	*N* = **16** Inpatient psychiatric wards Psychiatrist (*N* = 4); Psychologist (*N* = 4); Mental Health Nurse (*N* = 8)	Semi-structured interviews	Systematic text condensation and theoretically scrutinized	Satisfactory
Fletcher (1999) [68]	To identify the way nurses perceive the purpose, nature and meaning of constant observation.	UK	*N* = **12** Inpatient psychiatric wards Registered Nurses (*N* = 4); Enrolled Nurses (*N* = 2); Student Nurses (*N* = 2); Nursing Auxiliaries (*N* = 4)	Participant observations and interviews	Content analysis	Satisfactory
Nolan and Quinn (2012) [73]	To explore the reality of the everyday practice of mental health social work professionals in managing the risks service users with mental health issues face and present.	UK	*N*= **7** Community mental health teams Social workers (*N* = 7)	Semi-structured interviews	Grounded theory and the constant comparative method	Satisfactory

### Coder reliability and sensitivity analysis

Interrater agreement between the two coders across the three SDM components and 14 TDF domains ranged from 83.1 to 100%. For the sensitivity analysis, removing all the studies that gained an overall ‘satisfactory’ rating [60–64, 67–70, 73, 74] resulted in one domain (knowledge) no longer being relevant. The same salient TDF domains were identified, with the addition of ‘beliefs about consequences’ and ‘emotions’. The findings of the sensitivity analysis demonstrated that the exclusion of these studies would have had a small impact on the overall findings.

### Data synthesis

The following section begins by summarising study findings relating to the components of SDM. Then, the key barriers and enablers within each of the TDF domains and COM-B components are summarised.

#### SDM components

None of the included studies directly referred to the term SDM in RA and RM with individuals with mental illness. However, all studies reported on at least one component of the ‘Three Is of Influence’ SDM model [2].

The ‘informed’ component was identified in several of the included studies. Professionals spoke openly about not discussing risk with service users; that RA was undertaken without the service user’s knowledge; and that the content of the RA was not always shared with the individual [16, 20, 62, 63, 66, 81]. Conversely, in describing RM practices, professionals emphasised the importance of providing information to service users during observation and mechanical restraint [61, 71, 82]. In a study about forensic mental health services, professionals believed that keeping the service user informed and prepared before meetings, as well as discussing risk factors contributed to forming a trusting relationship [64].

In other studies, professionals acknowledged that they do not generally involve service users in the RA process [16, 20, 63, 64], some reported involving service users for obligatory, and information gathering purposes [20, 67, 70, 82]. Others believed it was important to involve and collaborate with service users in RM planning [64, 65, 83] for reasons discussed later.

The ‘influence’ component was also mapped to findings within this review. Some professionals described the need to make decisions on behalf of the service user [66, 70, 72, 83], thus inhibiting the service user’s influence in the RA and RM process. Other professionals valued collaborating with service users and supporting their choice in decisions that involved risk [60, 64]. Positive risk-taking was encouraged to support service users’ influence in decision-making [66, 71–73].

#### Barriers and enablers

Through the use of the TDF [52], potential barriers and enablers to the SDM components in RA and RM were identified. Barriers and enablers ranged across twelve domains: *knowledge, skills; social/professional role and identity; beliefs about capabilities; beliefs about consequences; reinforcement; intentions; goals; memory, attention and decision processes; environmental context and resources; social influences; and emotions*. Relevant domains, and the how they relate to barriers and enablers are presented in Table 3.

**Table 3:** TDF domain mapped to the barriers and enablers

TDF Domains	Themes	Awenat et al (2017) [81]	Barnicot et al (2017) [71]	Coffey et al (2017) [20]	Coffey et al (2019) [62]	Felton et al (2018) [66]	Fletcher (1999) [68]	Forsberg et al (2018) [60]	Gunstone (2003) [70]	Hagen et al (2017) [74]	Holley et al (2016) [72]	Langan (2008) [16]	Lees et al (2014) [69]	Nielsen et al (2018) [61]	Nolan and Quinn (2012) [73]	Nyman et al (2020) [64]	Sun et al (2006) [67]	Rimondini et al (2019) [65]	Vandewalle et al (2019a) [82]	Vandewalle et al (2019b) [83]	Woods (2013) [63]	No of studies by domain
**Knowledge**	*Policy or guidelines*																					**2**
**Memory, attention & decision processes**	*Type or level risk*																					**4**
	*Individual Factors*																					
**Skills**	*Training (or lack of)*																					**10**
	*Adapting Language*																					
**Social Influence**	*Risk Vs Recovery*																					**18**
	*Power or best interest*																					
	*Service user capacity/insight*																					
	*Risk averse team culture*																					
	*Therapeutic relationship*																					
	*Supervision*																					
**Environmental context and resources**	*Lack of staff, time, resources*																					**12**
	*Setting or meeting forum*																					
	*Local policies and procedures*																					
**Social professional role and identity**	*Not my role*																					**16**
	*My professional role and responsibility*																					
	*Decision shared with MDT*																					
	*Service user jointly responsible*																					
**Beliefs about capabilities**	*Difficult/sensitive topic*																					**11**
	*Lack of confidence*																					
	*Resolving disagreements*																					
	*Level of agreement*																					
**Beliefs about consequences**	*Fear of causing distress/harm*																					**10**
	*Disengagement*																					
	*Stigma and labelling*																					
	*Fear of blame/accountability*																					
	*Fear for personal safety*																					
**Intention**	*Acceptance of current practice*																					**4**
	*Aspiration*																					
**Goals**	*Not a priority*																					**13**
	*Obligatory reasons*																					
	*A shared decision*																					
	*To provide knowledge*																					
	*Improve RM or reduce risk*																					
**Reinforcement**	*Value collaboration or SDM*																					**14**
	*Positive risk-taking*																					
	*Promote empowerment or recovery*																					
	*Empathy or compassion*																					
**Emotions**	*Anxiety*																					**9**
	*Fear*																					

Shading represents a barrier,  represents an enabler

Reference: [42, 75–80]

**Fig. 2:**
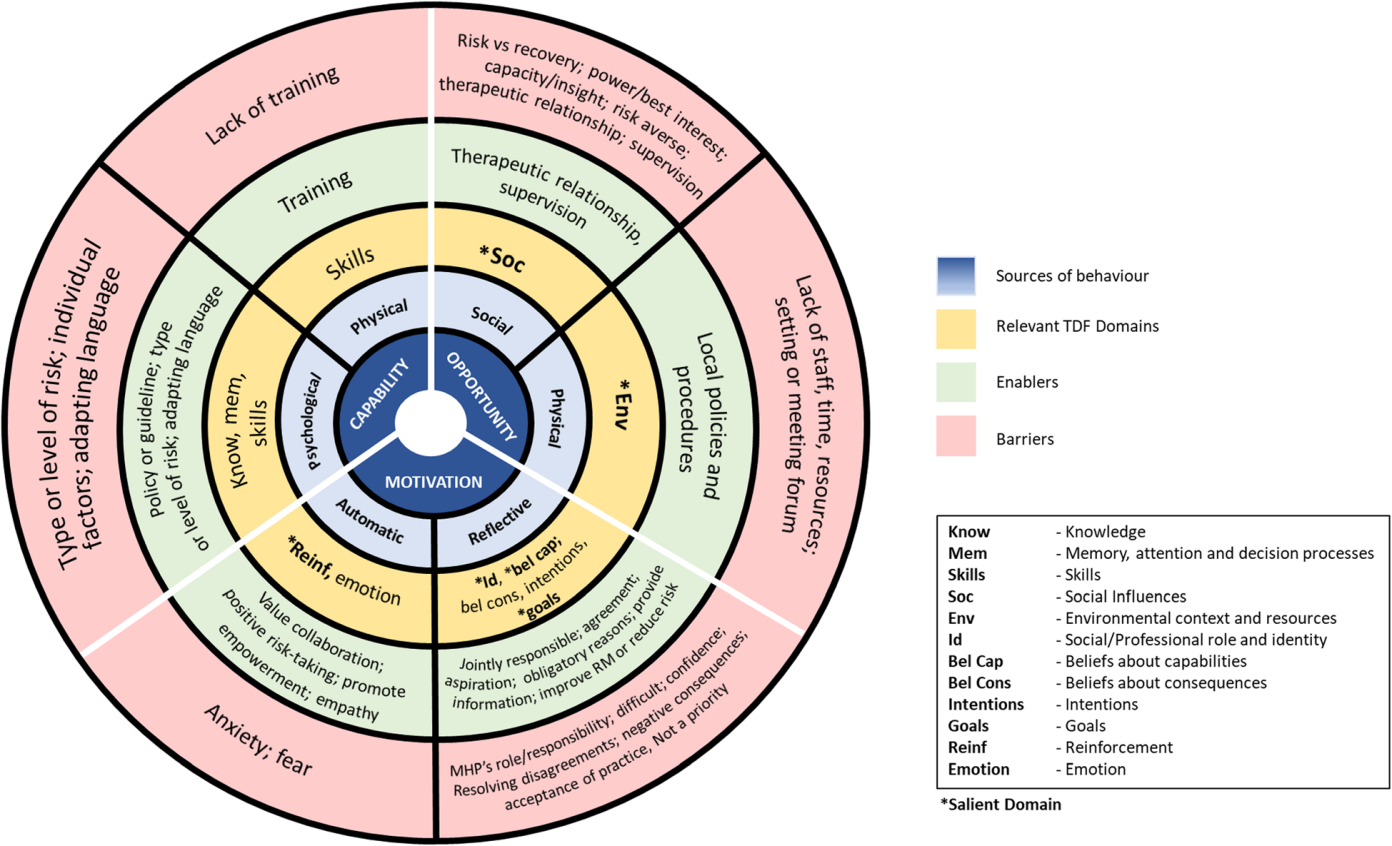
TDF domains mapped to COM-B components and sub-components

TDF domains (and the themes within) were then mapped to COM-B components and sub-components (Fig. 2). Based on a frequency count of studies by domain (Table 3), the most relevant domains were: social influences (*n* = 18); social/professional role and identity (*n* = 16); reinforcement (*n* = 14); goal (*n* = 13); environmental context and resources (*n* = 12) and beliefs about capabilities (*n* = 11). The key barriers were ‘power and best interest’ (*n* = 11) and ‘my professional role and responsibility’ (*n* = 12). The key enablers were ‘therapeutic relationship’ (*n* = 12), and ‘value collaboration’ (*n* = 11). The key barriers and enablers linked with TDF domains: ‘social influences’, ‘social/professional role and identity’ and ‘reinforcement’. The salient TDF domains (and barriers and enablers within) matched COM-B components: ‘opportunity’ and ‘motivation’.

Below, is a summary of the review findings of the barriers and enablers matched to TDF domain and COM-B component. Both first-order (direct quotations) and second-order (authors interpretation) themes are presented using illustrative quotations. Direct quotes have been presented in italics.

#### Capability

##### Knowledge

Professionals referred to policy and legislation in guiding them in supporting service users’ influence in decision-making or risk-taking [73]. In a study about service users who wish to discontinue taking neuroleptic medication [60], professionals working in early intervention services demonstrated openness towards supporting discontinuation and said that this was guided by their understanding of the National Institute for Health and Care Excellence (NICE) guidelines and research:

*“The evidence we have is that it is worth giving most people a trial off the medication in order to see if their illness would be a relapsing recurring one”* [60] p244)

##### Memory, attention and decision processes

Professionals’ implementation behaviours may have been influenced by the type of risk identified. In Langan [16], professionals believed that service users were less involved in a discussion about risk to others than risk to self:

*“I think risk to other people tends to be thought of as being...You know, look at it historically and see what has happened before. Whereas, risk of suicide, although that’s important as well, tends to be more on how the patient feels, in terms of harming themselves, at that time. So, probably, risk to self is more centred on the patient”* [16] p476)

In other studies, individual factors were key in determining service users’ readiness to be released from mechanical restraint [61]; and if risk-taking could be supported [60, 73].

##### Skills

Some professionals attributed their reluctance to discussing suicide with service users to lack of formal training [69, 74, 81, 83]*.* Limited training was also considered a barrier to engaging service users in RM:

*“I have never done any training on this topic. I know that I may change my attitude towards the patients, but I don’t know how to do it”* [65] p7)

Some professionals’ believed that additional training in risk would enhance their practice in caring for suicidal service users [69]. In a study about risk to others [16], a psychiatrist explained how training in RA and RM enabled him to discuss risk openly with a service user:

Professionals described adapting the language of risk to aid them in communicating with service users. In Langan and Lindow [42], professionals questioned the helpfulness in using the term risk: *“I mean, I don’t like to use terms like ‘risk’ in that sense, but I mean I think he does accept that there are concerns about his behaviour”* [42] *p16).* Instead, they reported using terms such as “early warning signs” or “relapse indicators” to facilitate discussion about risk with service users.

In a study about suicidal ideation, nurses reported adapting their communication to align with the service user’s communication preferences [82]:

*“I ask patients how they feel about it when I talk to them about suicidality and how they prefer to have these interactions”* [82] p2870)

Professionals also reported adapting their communication style with individuals who wished to discontinue taking neuroleptic medication [60]. The communication style that they adopted, i.e. collaborative or coercive, was based on their judgement of the risk factors and perceived outcome. Other professionals were reported to have used euphemistic language to avoid open dialogue about suicide with service users,*: “oh, well, you know, if you’re not feeling right”* [81] p105)).

#### Opportunity

##### Social influences

The tension between managing risk and promoting recovery resulted in professionals experiencing role conflict [20, 64, 72–74, 83]. Findings indicate that RM practices influence other aspects of care including therapeutic relationships, decision-making, and recovery [64, 65, 72, 74, 78]. In a study about continuous observation [71, 77], a professional explained that while developing a therapeutic relationship with the service user was important, the utmost priority was maintaining safety:

*“Every encounter with a patient should be made therapeutic … but it isn’t the primary purpose. The primary purpose is safety. I think the policy makes it very clear that safety trumps everything else”* [77] p553)

Findings suggest that the pressure of managing risk could lead to power imbalances that inhibit service users’ involvement or influence in the decision-making process:

‘ … risk dominated the decision-making of professionals to such an extent that it defined how service users were understood and treated with limited evidence of power-sharing and involvement of service users in decisions’ [66] p1142).

Some professionals reported using coercion [68] to maintain the service users safety:

*“If we indicate to patients that we are going to the seclusion room, then few patients say they’d “rather not”. But even when they say they’d “rather not”, we do it anyway, and then we emphasise, “Look, we want to protect you against your thoughts”* [83] p1129)

Decisions about risk are sometimes made by professionals in what they believe to be the service user’s best interest [16, 20, 60, 65, 66, 70–72, 74]:

*“Of course it can get difficult if the service user says no, “I want, I want to do it my way now,“ Um, and then you have to have a very different conversation and you need to say that we feel collectively as a team that at this stage it’s still a risk”* [72] p4)

Factors relating directly to the service user, such as insight or mental capacity impede on the SDM components in RA and RM [42, 60, 61, 65, 70–73, 83]:

*“We can share the responsibility with the patient only when he has totally understood and accepted what is happening to himself, otherwise it is very difficult …*” [65] p7)

A risk-averse team culture was highlighted as a barrier to positive risk-taking [72, 73] and the sharing of risk information with service users:

*“To my shame, there are cases that I follow that culture, that I hide that risk assessment or secret. Why? Because I want to protect the individual from the knowledge of that.., their illness that they have can be a risk to themselves or to the others. It’s a practice that I’m not very comfortable but nevertheless, I raise my hand and say I have”* [20] p6)

Some professionals’ reluctance to talk openly about suicide or trauma was reinforced in team culture [81, 83]. In a study about service users who wished to discontinue from neuroleptic medication, professionals spoke about the change in service culture [60]. With the ‘old’ culture described as less acceptant of discontinuation and service users influence in the decision-making process.

Developing a therapeutic relationship and trust enabled professionals to facilitate discussion about risk with service users [16, 69, 82], as well to collaborate in RM [71] and gather information for RA purposes [67]:

*“Rapport is key*. *.. it means I can get the information I need and that they’re more likely to actually tell me whether they’re still suicidal or not, and then from there we can work out what they need together”* [69] p310)

Others felt that knowing the service user enabled them to support positive risk-taking:

*“If you’re beginning to know a bit more about who they are, you might feel able to take greater therapeutic risks, in the hope of encouraging them to take responsibility”* [71] p478)

A good therapeutic relationship was reported to be beneficial in challenging situation, for example, communicating negative decisions to service users [64]. Therapeutic trust and alliance were also viewed as critical strategies in engaging service users in RM [61, 65].

Conversely, where the quality of the therapeutic relationship was less than ideal, it was considered a barrier to involving service users in RA and RM. Staff acknowledged that they were more likely to err on the side of caution with RM with service users that were less well known [71]. In other studies, professionals recognised that the therapeutic relationship may be better with one professional compared to another and that this could impact on the service user’s openness about risk and engagement in RM [61, 77]. Authors concluded that professionals lack of interaction with service users and distance from their subjective experience suggest a relational distance [66]. In a study about the risk to others, professional’s tentativeness in language, for example, *“I try to discuss risk with him”*, was attributed to the quality of therapeutic relationship [42].

Supervision was considered essential and beneficial to support discussing risk, such as suicidality, with service users [69, 81, 83]; and perceived as an enabler to engaging service users in RM [71, 77]:

##### Environmental context and resources

Professionals reported that they did not have the time or opportunity to get to know or directly relate to service users [65, 66, 71]. High caseloads, staff shortages, lack of training and resources were highlighted as factors that impede practice [63, 66, 69]. For example, in Forsberg, Tai [60], the pressure of increased caseloads, administration and service targets were reported as barriers to supporting service users to discontinue from medication. In a study about suicidal ideation, a nurse reported:

*“Sometimes I spend more time reporting than being present with the person. That is a shame! I sometimes wonder what is most important, “What I write down or what I really do with that person?”. Of course, I believe it is important that you write down things in case something happens, but I also believe that there are too many administrative tasks”* [83] p1130)

In Felton, Repper [66], professionals recognised that most of their time was spent in an office and that this caused a spatial distance between themselves and service users. Professionals were critical of organisational requirements to persistently document risks [82] and the amount of screening and assessments they needed to do for service users at risk of suicide. Instead, they questioned the value of these tasks as they believed it limited their time to meaningfully engage with service users.

Findings indicate that the setting or meeting structure used to discuss and make decisions about risk may impede on the service user’s involvement or influence in the process [62, 66].

“Formal ward round-based review meetings were named as a place for risks to be discussed although not necessarily in the presence of service users” [62] p12).

Nurses reported the difficulty in communicating risk with service users when they were not invited to the RA meeting or not directly involved in developing the RA [64], and they believed that this hindered their ability to promote the service users participation in decisions. Professionals also highlighted that if the environment or setting was inappropriate, for example unsafe, noisy and distracting, this could impact on the service users’ involvement in RM [65, 77].

Local policies and procedures were considered an aid to communication about risk with service users. In Langan [16], a voluntary sector organisation reported that their local policies encourage openness between professionals and service users about risk. Specifically, it was a requirement for professionals to complete RA forms jointly with service users, or the voluntary organisation operated an open access policy where individuals could freely access any information about their risks.

#### Motivation

##### Social/professional role and identity

Findings indicate that professionals retain responsibility for managing risk [16, 20, 63, 65, 66, 70–72, 74], which may be influencing the service users involvement in the RA and RM process.

Findings mapped to this domain were associated with data within the ‘social influences’ domain, for example, professionals making decisions in the best interest of the service user or conforming to their teams’ risk averse culture. In Holley, Chambers [72], professionals described making decisions on behalf of service users by drawing on their professional knowledge and expertise for managing risk.

In many of the included studies, decision-making regarding risk was described as a team responsibility with little mention of the service user’s input [66, 67, 70, 72]. In a study about service users who self-neglect, the author concluded that:

“it was not clear how often the teams made decisions based on what they thought was appropriate for the client, rather than on the client’s personal and informed choice” [70].

Professionals’ responsibility for reducing risk of harm to the individual and others conflicted with their intention to work collaboratively with the service user:

*“You know they [meaning colleagues] have a duty to protect the populous from risk. Sometimes that may not chime with the personal interest of the patient ...”* [60] p243)

Findings indicate that therapeutic engagement with individuals at risk of suicide was not always prioritised by nurses or realised by other MHP’s as part of their role [69, 81]. For some, facilitating discussion about suicidality or trauma was considered the responsibility of the psychologist or psychiatrist [81, 83]*.* For others, the service user was responsible for initiating discussion about suicidality:

*“Basically, it’s down to them to tell us … we’ve no other way really unless they already told their relative so they’re gonna have to be speaking about it”* [81] p105)

##### Beliefs about capabilities

Conversations with service users about risk and therapeutic risk-taking were described as difficult [16, 66, 81]. Some professionals lacked confidence in approaching the topic of ‘risk to others’ with service users [16], whereas others expressed a lack of confidence about how to talk with service users about suicide [81]. Professionals highlighted the need for more training on suicidality in their education:

‘ … although all participants are specialized in mental health nursing, one of them stated that she does not feel educated or confident enough to talk with patients about suicide, and another informant stated that there should be much more focus on caring for suicidal persons in the education’ [80] p33).

They acknowledged that risk information might not be shared with service users because of potential disagreements [20]. In a study about the risk to others, reaching a mutual agreement with an individual who disagreed with their identified risks was described as challenging:

*“Very difficult. Very difficult. He’ll deny many of the incidents that I’ve told you about. He’ll say that the police are wrong, that they were harassing him. That he didn’t do these things. That he’s not a risk to other people …. So it’s very, very difficult, yeah, to find any middle ground there really”* [42] p18)

When the service user and professional had conflicting viewpoints about discontinuation from medication, this impeded on the service user’s influence in the process [60]. The professional, instead, attempted to increase the service user’s agreement with their perspective.

On the other hand, the level of agreement about risk was highlighted as an enabler to involving service users in RM:

*“Obviously, if they can acknowledge that there is a problem then we’re in a much better position to ensure that they put something in place which works”* [42] p17)

##### Beliefs about consequences

Professionals expressed a range of views about the potential consequences of involving service users in the RA and RM process. Many were concerned that discussing risk with a service user or involving them in RM would cause the individual distress or harm [16, 20, 81, 82]:

*“Sometimes we avoid involving patients in order to preserve his saneness. In the psychiatric field is difficult to evaluate how much information the patient may tolerate”* [65] p7)

Some professionals believed that discussing risk with others could be damaging to their therapeutic relationship with the service user and lead to disengagement [16]*.* Others were worried that involving service users in RA would reinforce stigma:

*“the stigma of the mental health is still very prevalent in our society so by doing a risk assessment you more or less emphasise that stigma. .. You are a very risky person, you’re dangerous to yourself, and you’re dangerous to society, whereas this doesn’t go well with the recovery that we try to achieve for that person”* [20] p8)

Professionals also feared negative consequences for themselves by discussing risk with service users. In Awenat, Peters [81], following a suicide, professionals were worried about being blamed for negligence. This resulted in them recording detailed information to clear themselves of blame should a suicide occur, as well as cautious discussions with service users in case they disclosed suicidal ideation. Similarly, in other studies, professionals highlighted the need to document decisions accurately and follow protocol to protect themselves from blame should their decision be questioned [74, 83]. Professionals who encouraged risk-taking [73] or supported a service user’s wish to discontinue from medication [60] were also fearful of being blamed if negative outcomes occurred as a result of their decision.

*“Risk-taking and promoting an individual’s freedom is encouraged but you’re conscious of the fact that if someone gets hurt, it’s not just them. .. criticism will be levelled at each level within the authority”* [73] p180)

In other studies, fear of being blamed influenced the decision-making process and resulted in professionals adopting defensive or restrictive approaches [71, 83].

Professionals’ concern for their personal safety acted as a barrier to both discussing ‘risk to others’ with service users [16] and involving service users in RM [65].

##### Intentions

Some professionals were resigned to their current practice of not involving service users in the RA and RM process [20]. Others were willing to move towards involving service user more in the process:

*“I’m quite open to change and including the person more in it, rather than it just being professionals talking about the risks”* [16] p477)

Nonetheless, professionals’ aspirations for greater service user involvement in RA and RM did not necessarily reflect practice [72]:

‘Whilst everyone considered openness a good idea in principle, practice had not always caught up with aspirations’ [16].

##### Goals

The extent to which professionals consider the SDM components important in the RA and RM process influenced their implementation behaviour. For example, involving service users in RA and RM was not considered a priority for some professionals:

‘… they had given little consideration to how they could directly and actively involve clients in the assessment and management of risk’ [63] p810).

For others, interpersonal engagement with service users at risk of suicide was not prioritised [69] and discussion about suicidal ideation was considered counterproductive [68]. Obligatory reasons for involving service users in RA and RM practices, i.e. for assessment and information gathering purposes, were provided by professionals in several studies [20, 61, 63, 65, 67, 70, 74, 82, 83]:

*“In order to take care of these suicidal patients, I try to build a trusting relationship with them. If I can build a good trusting relationship with them, they will trust me. They will give me the information I need and then we can explore their problems and try to help them to prevent future suicide attempts”* [67] p687)

Forming agreements with service users (or a shared-decision) was considered an important step in the RM process [61, 82, 83]. In several studies, professionals emphasised the importance in openly communicating about risk, as well as providing the service user with knowledge and information about their risk [16, 65, 71, 83]:

‘These nurses avoid imposing instant protection and instead engage in dialogue with patients that facilitates understanding of risks and potentially risky situations (e.g. taking a bath), the meaning that patients attach to risks and potentially risky situations, and what can be done to address risks’ [83] p1126).

Professionals acknowledged that RM was more likely to be helpful or effective if the service user was involved in the RA process [16, 61, 65, 67, 69, 71, 82, 83]:

*“I think it’s more of a risk if it’s other people talking about them behind their back. I think the more that things can be out in the open, the less of a risk it is”* [42] p14)

##### Reinforcement

Professionals emphasised the importance in communicating to service users about their risk [72], as well as encouraging service users to talk about their distress or suicidality [81–83].

*“The opportunity to interact is the ultimate. .. it’s a really important interaction..*. *It can be the difference between life and death”* [69] p309)

Some believed that RM was more likely helpful if service users were involved in decision-making [71]. Others valued supporting choice and collaboration, and this guided their interaction with service users who wished to discontinue from medication [60]. Positive risk-taking encouraged some professionals to support the service user’s choice or influence [61, 62, 71–73].

Professionals were motivated to support service users’ influence and positive risk-taking as this favoured autonomy, empowerment, and recovery [65, 66, 72, 73, 82]:

*“if it is her wish to look after her finances then actually she is entitled and that needs to be explored very slowly with her [. . .] You can give her advice whether it’s a good decision or a bad decision but it’s her decision to take control of it”* [72] p3)

Professionals stressed the importance in demonstrating empathy, compassion and instilling hope [67, 69, 77, 82, 83]. They believed that empathy supported service user to work through their distress and talk about suicidal feelings:

*“I feel it’s important to feel and show empathy. If you don’t have empathy, you have no way of realising the patients’ torment and discomfort, or how serious or how strongly they feel about attempting suicide”* [67] p687)

##### Emotions

Professionals expressed negative emotions that impact on the assessment and management of risk with individuals with mental illness. In Barnicot, Insua-Summerhayes [71], anxiety in preventing harm and about being blamed may have influenced decision-making around continuous observation and led to restrictive practices. The possibility of a negative outcome from supporting a service user to discontinue from medication triggered anxiety in professionals [60]. While approaching the issue of risk created anxiety for some professionals [20, 66, 80], others expressed fear in approaching sensitive topics such as risk to others [16] or suicidal risk [69, 80, 81]. For example, a professional described their concern about possibly being the last person to have spoken to someone who takes their own life:

*“I think it’s scary because you don’t want to be the last person having that conversation and they do something. You don’t want to think you’ve done anything that could have erm, actually aggravated them or tipped them over the edge or you’ve said something that has made them think about something”* [81] p106)

## Discussion

The findings of this review indicate that SDM is not a term commonly used in mental health services when exploring processes of RA and RM. The components of SDM (i.e. informed, involved and influential) are referred to but are not being implemented consistently in the RA and RM process. MHPs spoke openly about not discussing risk with service users, involving service users in the process, or supporting their influence in decision-making about risk. This is in line with studies of service user accounts of RA and RM [20, 38, 42], where it was found that service users were often unaware of the RA and RM plan.

Through the use of the TDF [52], this systematic review has provided a comprehensive understanding of the perceived barriers and enablers to the SDM components in RA and RM from the literature. The salient COM-B components (and linked TDF domains) identified from the findings of this review were social and physical opportunity (i.e. ‘social influences’ and ‘environmental context and resources’), which refer to the social, cultural, and environmental influences on behaviour; and reflective and automatic motivation (i.e. ‘social/professional role and identity’, ‘beliefs about capabilities’, ‘goals’ and ‘reinforcement’), which characterise the cognitive processes that drive behaviour.

Mental health policy at an international level recommends that the processes of RA and RM are collaborative, person-centered and based on SDM [28, 33, 84]; however, there were many factors identified in this review that potentially impede on practice.

Managing risk and delivering recovery-orientated care were experienced as competing priorities that led to practice dilemma. The tension was believed to arise from organisational expectations, legal responsibilities, and contradictory frameworks of practice. Policy guidelines emphasise protection, harm minimisation, public safety, and duty of care. At the same time, they recommend recovery-orientated care based upon the components of SDM, positive risk-taking, therapeutic relationships, and empowerment. Our findings show professionals acknowledged the primacy of RM and the impact this had on other aspects of care including therapeutic relationships, and positive risk-taking. Boardman and Roberts [37] argue that it is possible to strike a balance between managing risk and delivering recovery-orientated care. They propose shifting towards a ‘person-centred’ approach to assessing and managing risk, based on SDM and collaborative safety planning.

Reluctance to talk about suicidality with service users or to support positive risk-taking were believed to be reinforced in a risk-averse team culture. Simpson [85] reported similar findings and highlighted the need for a ‘safe’ environment for professionals to openly discuss and disclose uncertainties, challenges, and alternative treatment options within the team. In addition, the findings of this review suggest that professionals tried to make decisions about risk with the service users’ best interests in mind, but at times this was the professionals’ interpretation of best interests and not necessarily the service users’. This is problematic as a capacitous service user is the expert on their own best interests, and even when not capacitous their wishes and views ought to be taken into account. Factors relating directly to the service user, such as capacity and insight, were considered barriers to discussing risk and collaborating with the service user in RM planning, thus impeding best interest decisions. It has been argued that paternalistic approaches to decision-making can cause practice conflicts between the ethical principles of autonomy on the one hand, and beneficence and non-maleficence on the other [86]. In mental health care, decision-making can be justified in terms of respecting the service user’s choice (autonomy), the professional’s duty to promote good (beneficence) or to prevent harm (non-maleficence) [86]. Paternalistic approaches may conflict with the autonomy of a non-capacitous service user, when decisions are made based on the professional’s interpretation of the best interests of the service user [87]. Experiencing a mental health crisis can lead to diminished capacity and competency to make a decision and in these circumstances, paternalistic interventions have been justified on the basis of the requirements of beneficence or non-maleficence [88]. Breeze [87] argues that the assessment of rationality or competency has the potential to be subjective and value-laden and although paternalism maybe justified in some situations, it should be exercised with caution. For example, where there is a disagreement between the professional and service user about what is considered ‘best interest’, it should not be assumed that the service user’s view is irrational or wrong, indeed S. 1 [4] Mental Capacity Act (2005) states that *‘A person is not to be treated as unable to make a decision merely because he makes an unwise decision’* [89].

Developing a therapeutic relationship and gaining trust enabled professionals to engage service users in a discussion about suicidality, as well as promote positive risk-taking and collaboration in RM. A recent review of service users’ perspectives of helpful RM practices [43] found that interpersonal relationship and communication aided RM to be inclusive for service users, and trust was considered to nurture open discussion about risk. In a study about risk-taking and recovery [90], service users also reported that therapeutic relationships developed trust, and this led to more collaborative discussion and decision-making.

Study findings suggest that professionals may be retaining responsibility for assessing and managing risk and thus limiting the extent to which service users are genuinely informed, involved or influential in the process. Negative beliefs about consequences inhibited professionals from implementing SDM in RA and RM. On the one hand, professionals were concerned that discussing risk could cause the service user distress, to disengage from services or to feel stigmatised. On the other hand, professionals were fearful of being blamed or investigated for negative outcomes from supporting risk-taking, i.e. service user who wished to discontinue taking medication, or discussing suicidality. Fear of blame led professionals to accurately document decision-making to protect themselves should their decision later be questioned, as well as cautious discussion with service users about suicidal thoughts. A culture of blame and risk aversion continues to pervade mental health services [91] that is said to derive from bureaucratic management styles, perception of failure, political pressures and media influences [17, 92]. In a qualitative study, professionals expressed concern about restrictive practices potentially being eliminated as they felt that this would make it difficult to maintain safety [93], they were also concerned about being blamed when a negative event occurred.

Beliefs about consequences provoked negative emotions for some professionals who expressed fear and anxiety about preventing harm. Supervision was highlighted as a potential aid in discussing suicidal thoughts with service users. Tragic incidents can occur even after careful decision-making and thus professionals can expect to be accountable for decision-making and its implementation but not outcomes that they have no control over [94]. For MHPs to move away from paternalism and towards promoting SDM, change needs to occur at an organisational level [37]. Professionals need to know that they have managerial and institutional support, especially in situations where negative beliefs about consequences occur. It has been suggested that developing therapeutic risk-taking in practice requires organisations to support professionals by creating safe spaces to hold uncertainty, multidisciplinary working, shared responsibility, and supervision [88]. Institutional fear of things ‘going wrong’ is perhaps not helped by anxieties over the hyperbolic media coverage that can emerge when tragedies do occur [95]. The media’s negative portrayal of mental illness and misleading association with violence [96, 97] may contribute to the continuing stigma of mental illness; the preoccupation with RM in mental health care; and misconstrued perceptions of the actual risk posed towards others by individuals with mental illness. In reality, 11% of all homicide convictions in the UK, during 2007–2017, were patient homicides, i.e. people in contact with mental health services in the 12 months prior to the offence [98].

A lack of confidence in discussing certain types of risks with service users was reported. For example, professionals expressed concern about approaching the topic of ‘risk to others’, and uncertainty in how to initiate discussions about suicide with service users. In mental health care, it is recognised that RA and RM practices focus on ‘dramatic risks’ that involve harm to self or others [37], however, these extreme harms relate to a minority of people in contact with mental health services [98]. Dixon [38] compared service users’ and professionals’ ratings of risk and found that service users identified more risks in relation to their vulnerability, such as self-neglect and suicide, than professionals did. In contrast, professionals identified more risks than service users in relation to risk of harm to others. A collaborative safety planning approach would broaden the focus on risk to include the service users perspectives and consideration of everyday risks that are common but less considered in the assessment and management of risk [37]. Changing the language of risk and basing discussions on safety-concerns offer an alternative way of involving service users’ in managing their own safety and opens discussion about risk [99].

In the current review, professionals questioned their ability to resolve disagreements with service users about risk to others. Consequently, conversations about risk with service users were described as difficult. A systematic review of services users’ perceptions of RM found that people’s desire for honesty and collaboration was fulfilled when they felt listened to, despite disagreements. Furthermore, some services users recognised disagreements as an authentic part of therapeutic relationships [43].

As found in the broader recovery-focused care-planning and coordination literature [75], high caseloads, staff shortages and a lack of resource were highlighted as factors that impede on practice. Professionals reported limited time or opportunity to support positive risk-taking or to meaningfully engage with service users. Also, insufficient training on RA and RM negatively impacted on professionals’ ability to talk openly about risk. In one of the included studies, a professional who had received RA training reported that it enabled him to face his fear in discussing risk openly with an individual who had previously damaged his office [16]. Higgins, Doyle [24] research findings indicate the need for training to enable professionals to adopt a collaborative RA and safety planning approach. They propose training delivered at undergraduate and postgraduate level that includes the skills necessary to engage service users and carers in the RA and safety planning process [24].

Professionals’ behaviours were guided by their perceived outcomes of implementing the SDM components in RA and RM. For some professionals, involving service users in RA and RM was not always a priority. Others, however, were motivated to involve service users for obligatory reasons, as well as to provide the service users with knowledge and understanding of risks and to collaborate in reducing risks. Similar to the findings of Kaminskiy, Senner [12] qualitative synthesis, this review found support from MHPs for the idea of implementing SDM or working in collaboration with service users. Professionals’ emphasised the importance in communicating risk with service users, promoting empowerment and demonstrating empathy. Some described adjusting their language to facilitate discussions about risk, while others expressed aspiration towards involving service users in future RA and RM practices, though it was recognised that aspiration may have not yet influenced practice.

### Strengths and limitations

This is the first systematic review of evidence reporting MHPs’ experiences and attitudes towards SDM in RA and RM, which uses both the TDF and COM-B model to synthesise findings. The synthesis was informed by several psychological theories of behaviour change and empirical findings of included studies. However, this review is not without limitation. First, the review focused on MHPs’ experiences of SDM in RA and RM: thus, the service users’ perspective was not examined, however, a recent mixed-studies systematic review explored helpful RM practices from the service users’ viewpoint [43]. Secondly, despite conducting systematic searches, SDM is not a well-indexed term, and researchers have varying interpretations of the concept: therefore, our search strategy may have inadvertently missed relevant studies. To capture relevant studies in our searches, we used MeSH terms for SDM and included additional free text key terms related to the concept of SDM (e.g., service user involvement, patient-centred and recovery). Thirdly, it is important to note that the decision to conduct a qualitative systematic review was derived from the findings of a scoping search, which indicated that qualitative methods dominated this field of research. A quantitative survey study [24] was identified, however, but excluded on the review’s eligibility criteria. Although the key focus of Higgins, Doyle [24] study was to explore mental health nurses’ practices and confidence in RA and safety planning, there was a small amount of data relevant to the findings of this review (i.e. stakeholders’ involvement in the RA and RM process). Lastly, the wide variation in methods employed in qualitative research poses challenges in the assessment of quality and synthesis of findings for the purpose of a review [49, 100]. Indeed, the present review included studies that differed significantly in design, data collection, and analysis method. Also, qualitative research is often criticised for lack of generalisability. Therefore, the strength of recommendation that can be made from the evidence included in this review is limited. Future reviews may wish to further develop the themes identified in this review by sourcing data from quantitative work.

## Conclusion

The findings of this review indicate that there may be limited SDM in RA and RM with individuals with mental health problems. Langan and Lindow [42] reported this over 15 years ago, and despite policies endorsing SDM it, largely, is not happening. This review identifies some of the key issues that may be underpinning this lack of action and warrant further intervention and investigation.

Through the use of the TDF and COM-B model, this review explored MHPs’ perceived barriers and enablers to SDM in RA and RM. Key barriers were ‘power and best interest’ and ‘my professional role and responsibility’, whereas key enablers were ‘therapeutic relationship’ and ‘value collaboration’. These barriers, enablers and TDF domains matched COM-B components ‘opportunity’ and ‘motivation’.

The finding from the present study contributes to existing knowledge of SDM by providing insight into MHPs’ perceived barriers and enablers to implementing SDM in RA and RM. Consistent with a qualitative synthesis study that examined attitudes towards SDM in the broader field of mental health [12], a lack of capacity was identified as a barrier to SDM in RA and RM. Although justified in some situations, mental capacity fluctuates with time and research indicates that most psychiatric in-patients are capable of making key treatment decisions [101]. There are also methods that can be used to incorporate service users’ views, such as decision aids, advance directives and advocacy. Therefore, diminished capacity alone should not be reason to exclude the service user from the RA and RM process, as the service user may still be able to offer valuable insight into their perspective and experiences with risk that can inform the RM plan. The present study also highlights the importance of the therapeutic relationship in facilitating discussions about risk with service users, which corroborates findings from a previous systematic review of service users’ perspectives of RM [43]. Therefore, increasing professionals’ opportunity to develop the therapeutic relationship may influence their motivation to implement SDM in RA and RM.

The findings of this review highlight a complex range of social, cultural and environmental factors that together influence SDM in RA and RM. This information will be relevant to policymakers and practitioners and can also be used to develop targeted interventions aimed at changing practice in this challenging area. However, these findings are based on a small number of studies that are heterogeneous in aim and objective. Furthermore, none of the included studies directly investigated SDM in RA and RM with individuals with mental illness. Therefore, further extensive work is needed to better understand how best to implement SDM in RA and RM so that all parties feel comfortable. A qualitative study by the lead author, directly investigating the barriers and enablers to SDM in RA and RM, is currently underway and has been developed from the findings of this review. The benefits of implementing SDM in RA and RM planning is also insufficiently researched. It is important to build an evidence base on the impact, as well as the acceptability and feasibility of a collaborative approach.

## Supplementary Information

**Additional file 1.**

**Additional file 2.**

**Additional file 3.**

## Data Availability

Data sharing is not applicable to this article as no datasets were generated or analysed during the current study.

## Abbreviations

SDM: Shared Decision Making
MHP: Mental Health Professional
RA: Risk Assessment
RM: Risk Management
DH: Department of Health
PRISMA: Preferred Reporting Items for Systematic Reviews and Meta-Analyses
PROSPERO: International prospective register of systematic reviews
SPIDER: Sample, Phenomenon of Interest, Design, Evaluation, Research type
BASE: Bielefeld Academic Search Engine
TDF: Theoretical Domains Framework
COM-B: Capability, Opportunity, Motivation to Behaviour
UK: United Kingdom
