# Supplementary material for: Mental health professionals’ perceived barriers and enablers to shared decision-making in risk assessment and risk management: a qualitative systematic review
Source: BMC Psychiatry. 2021 Nov 25;21:594. doi: 10.1186/s12888-021-03304-0 (PMC8613998; doi:10.1186/s12888-021-03304-0)
Supplement: Supplementary file 1 — Additional file 1. [file 12888_2021_3304_MOESM1_ESM.docx]

**Additional file 1:** Medline search string

1. exp Mental Health/

2. *mental disorders/ or *anxiety disorders/ or *"bipolar and related disorders"/ or exp mood disorders/ or exp neurotic disorders/ or "schizophrenia spectrum and other psychotic disorders"/ or *"trauma and stressor related disorders"/ or *stress disorders, traumatic/ or exp Psychotic Disorders/

3. *Mental Health Services/ or *Psychiatric Department, Hospital/ or *Hospitals, Psychiatric/

4. ((mental* or psychiatric) and (health or disorder* or ill* or disease* or condition* or disability or instability or problem*)).t

5. ((severe adj2 mental) or 'severe* mental* ill' or (psychol* adj2 (health or problem* or disorder* or ill*))).tw.

6. 1 or 2 or 3 or 4 or 5

7. *Health Personnel/ or *occupational therapy/ or *psychiatry/ or *psychiatric nursing/ or social work, psychiatric/

8. (professional* or 'mental health professional' or MHP or 'healthcare professional*' or worker* or staff* or 'care coordinator' or practitioner* or provider* or manager* or clinician* or physician* or psychiatrist* or doctor* or nurse or 'occupational therapist' or 'social worker' or psychologist* or GP).tw.

9. 7 or 8

10. *attitude/ or *"attitude of health personnel"/

11. (experience* or attitudes* or perspective* or opinion* or view* or belief* or knowledge or discourse* or awareness or implement or behavio?r* or practice*).tw.

12. 10 or 11

13. exp Decision Making/

14. exp Patient Participation/

15. ('shared decision making' or 'decision aid' or 'decision process' or 'decision support').t

16. (Shar* adj2 information).tw.

17. ((Patient or user or client or consumer or person) and (Invol* or led or centred or participation or satisfaction or choice or preference or understanding or discuss or dialog)).tw.

18. (Collabor* or Partnership or choice or behavio?r or Holistic care or Recov*).tw.

19. ((Joint* adj2 (working or deci*)) or (Recovery adj2 (orientated or care))).tw.

20. 13 or 14 or 15 or 16 or 17 or 18 or 19

21. *Risk Assessment/

22. *Risk Management/

23. ('risk assessment*' or 'risk management').tw.

24. (risk adj3 (assess* or manag*)).tw.

25. ((safety or crisis) and plan*).tw.

26. (communication adj3 risk).tw.

27. 21 or 22 or 23 or 24 or 25 or 26

28. 6 and 9 and 12 and 20 and 27
