# Supplementary material for: Mental health professionals’ perceived barriers and enablers to shared decision-making in risk assessment and risk management: a qualitative systematic review
Source: BMC Psychiatry. 2021 Nov 25;21:594. doi: 10.1186/s12888-021-03304-0 (PMC8613998; doi:10.1186/s12888-021-03304-0)
Supplement: Supplementary file 2 — Additional file 2. [file 12888_2021_3304_MOESM2_ESM.docx]

**Additional file 2:** *Definition of TDF constructs by domain (Cane et al., 2012), and application to review question (adapted from Nathan et al. (2018); Definition of SDM components (Stacey et al., 2015) and application to risk assessment and risk management*

| **Domain** | **Constructs** | **Application to review question** | **Examples/ Rules** |
| --- | --- | --- | --- |
| 1. Knowledge  (An awareness of the existence of something) | Knowledge (including knowledge of condition /scientific rationale), Procedural knowledge, Knowledge of task environment | Mental health professional’s awareness and familiarity with guidelines/policies that recommend implementing shared decision-making in risk assessment or management | - Policies - Guidelines - Recommendations |
| 2. Skills  (An ability or proficiency acquired through practice) | Skills, Skills development, Competence, Ability, Interpersonal skills, Practice, Skill assessment, Coping strategies | Training, skills and practice in implementing shared decision-making in risk assessment or risk management | - Training and skills |
| 3. Social/professional role and identity  (A coherent set of behaviours and displayed personal qualities of an individual in a social or work setting) | Professional identity, Professional role, Social identity, Professional boundaries, Professional confidence, Group identity, Leadership, Organisational commitment | The extent that implementation of shared decision-making in risk assessment or risk management is perceived as part of the mental health professional’s role | - Perceived as part of the professional’s role/responsibility (or not) - Perceived as a joint responsibility with service user |
| 4. Beliefs about capabilities  (Acceptance of the truth, reality, or validity about an ability, talent, or facility that a person can put to constructive use) | Self‐confidence, Perceived competence, Self‐efficacy, Perceived behavioural control, Beliefs, Self‐esteem, Empowerment, Professional confidence | The mental health professional’s confidence in implementing shared decision-making in risk assessment or risk management | - Confidence/ lack of confidence - Resolving disagreements or level of agreement as influencing factors - Difficult or sensitive   If it is the mental health professionals’ perception of another person or group (i.e. the service users/carers) regarding their skills, confidence, knowledge, ability, commitment then code as “Social influences” NB: If they are referring to their own “group” i.e. mental health professionals’ code as “Beliefs about capabilities”. |
| 5. Optimism  (The confidence that things will happen for the best or that desired goals will be attained) | Optimism, Pessimism, Unrealistic optimism, Identity | The mental health professionals’ confidence that in the future they will be able to implement shared decision making in risk assessment or risk management |  |
| 6. Beliefs about consequences  (Acceptance of the truth, reality, or validity about outcomes of a behaviour in a given situation) | Beliefs, Outcome expectancies, Characteristics of outcome expectancies, Anticipated regret, Consequents | The mental health professional’s belief about benefits/disadvantages of implementing shared decision-making in risk assessment or risk management | - Blame and accountability - Disengagement - Fear of causing upset or alarm - Negative/positive outcomes - Stigma and labelling |
| 7.Reinforcement  (Increasing the probability of a response by arranging a dependent relationship, or contingency, between the response and a given stimulus) | Rewards (proximal / distal, valued / not valued, probable / improbable), Incentives, Punishment, Consequents, Reinforcement, Contingencies, Sanctions | Factors that encourage/discourage professionals from implementing shared decision-making in risk assessment or risk management. | - Positive risk taking - Promote empowerment or recovery |
| 8. Intentions  (A conscious decision to perform a behaviour or a resolve to act in a certain way) | Stability of intentions, Stages of change model, Trans-theoretical model and stages of change | The mental health professional’s intention to implement shared decision-making in risk assessment or risk management | - Acceptance of practice - Aspiration - Avoidance |
| 9. Goals  (Mental representations of outcomes or end states that an individual wants to achieve) | Goals (distal / proximal), Goal priority, Goal / target setting, Goals (autonomous /controlled), Action planning (with relation to their intention to implement | The relative importance to mental health professionals of implementing shared decision-making in risk assessment or risk management | - Priority/not a priority - Obligatory purposes - Safeguard/protect service user from knowledge of risk - Provide service user knowledge or understanding of risks |
| 10. Memory, attention and decision processes  (The ability to retain information, focus selectively on aspects of the environment and choose between two or more alternatives) | Memory, Attention, Attention control, Decision making, Cognitive overload / tiredness | The extent to which implementing shared decision-making in risk assessment or risk management is part of regular practice  Decision making processes i.e. rules of thumbs used to decide whether or not to implement shared decision-making in risk assessment or management | - Type of risk (i.e. risk to others or risk to self) - Level of risk |
| 11. Environmental context and resources  (Any circumstance of a person's situation or environment that discourages or encourages the development of skills and abilities, independence, social competence, and adaptive behaviour) | Environmental stressors, Resources / material resources, Organisational culture /climate, Salient events / critical incidents, Person x environment interaction, Barriers and facilitators | The environmental context/situation that may encourage/discourage implementation of shared decision-making in risk assessment or risk management | - Bureaucratic demands (time, resources, staffing, caseloads) - Risk assessment/management process) - Meeting forum |
| 12. Social influences  (Those interpersonal processes that can cause individuals to change their thoughts, feelings, or behaviours) | Social pressure, Social norms, Group conformity, Social comparisons, Group norms, Social support, Power, Intergroup conflict, Alienation, Group identity, Modelling | The interpersonal relationships/process that may influence implementation of shared decision-making in risk assessment or risk management | - Power - MDT support - Risk vs recovery - Factors relating to the service user i.e. mental capacity, insight, understanding, willingness to discuss - Team culture - Supervision - Therapeutic relationship with service user |
| 13. Emotions  (A complex reaction pattern, involving experiential, behavioural, and physiological elements, by which the individual attempts to deal with a personally significant matter or event) | Fear, Anxiety, Affect, Stress, Depression, Positive / negative affect, Burn‐out | Mental health professionals’ emotions when implementing shared decision-making in risk assessment or risk management | - Anxiety - Fear - Dissatisfaction - Embarrassment/shame |
| 14. Behavioural regulation  (Anything aimed at managing or changing objectively observed or measured actions) | Self‐monitoring, Breaking habit, Action planning (with relation to monitoring their habits) | Mental health professionals’ ability to self-monitor and action plan to implement shared decision-making in risk assessment or risk management | - If the professional wanted to increase shared decision making in risk assessment and risk management in future, how would they? - Intervention suggestions |
| **Shared decision-making component** | **Definition** | | **Application to RA and RM** |
| Informed | Being informed refers to the practice of ensuring that service users, carers and professionals know what is available for consideration. Service users are experts by experience and professionals are experts in, for example, different treatment options, services and resources, and insights into the structure and organisational culture of health services.  Being informed entails genuinely valuing the significance of all information and having an understanding of the rationale for decisions. | | - Risk assessment and management being undertaken without the service user’s knowledge - Risk not discussed with service users - Service users unaware of their risks - Lack of openness about risk - Also vice versa, if risk is discussed with service users i.e. service user is informed of risks and professionals are open with service users about risks |
| Involved | Being involved entails being willing to adapt decisions in light of the information shared. Thus, all parties to decision-making processes should respond to the expertise of others to reach decisions.  This is more than just consulting with the service user but power sharing i.e. where the expertise of the service user is valued and where the service user is also an active participant in the decision-making process | | - Service users being involved for obligatory purposes - Risk being discussed with service e.g. informally - Lack of power sharing |
| Influential | Being influential in decision making entails considering and respecting other people’s views, even if they are not held by the majority.  For service users, having influence means genuinely holding power and accountability for decisions.  This may challenge healthcare professionals to support service users’ choices that are perceived as risky or ‘bad’. | | - Decision regarding risk being made on behalf of the service user - Professionals maintaining responsibility for risk decisions - Decision made without the service users informed choice - Positive risk taking. Service users supported to make decisions. |

**Application to review question adapted from definitions provided in** (Nathan et al., 2018)

CANE, J., O’CONNOR, D. & MICHIE, S. 2012. Validation of the theoretical domains framework for use in behaviour change and implementation research. *Implementation Science,* 7**,** 1.

NATHAN, N., ELTON, B., BABIC, M., MCCARTHY, N., SUTHERLAND, R., PRESSEAU, J., SEWARD, K., HODDER, R., BOOTH, D. & YOONG, S. L. 2018. Barriers and facilitators to the implementation of physical activity policies in schools: a systematic review. *Preventive Medicine,* 107**,** 45-53.

STACEY, G., FELTON, A., HUI, A., STICKLEY, T., HOUGHTON, P., DIAMOND, B., MORGAN, A., SHUTT, J. & WILLIS, M. 2015. Informed, involved and influential: three Is of shared decision making. *Mental Health Practice,* 19**,** 31-35.
