# Supplementary material for: Mental health professionals’ perceived barriers and enablers to shared decision-making in risk assessment and risk management: a qualitative systematic review
Source: BMC Psychiatry. 2021 Nov 25;21:594. doi: 10.1186/s12888-021-03304-0 (PMC8613998; doi:10.1186/s12888-021-03304-0)
Supplement: Supplementary file 3 — Additional file 3. [file 12888_2021_3304_MOESM3_ESM.docx]

**Additional file 3.** *Quality appraisal of included studies 1-10*

| Prompt | Reviewer | Coffey et al. (2016) | Gunstone (2003) | Holley et al (2016) | Langan (2008) | Woods (2013) | Barnicot et al (2017) | Felton et al (2018) | Awenat et al (2017) | Sun et al (2006) | Forsberg et al  (2018) |
| --- | --- | --- | --- | --- | --- | --- | --- | --- | --- | --- | --- |
| Are the research questions clear? | **1** 2 | **Y**  Y | **Y**  Y | **Y**  Y | **Y**  Y | **Y**  Y | **Y**  Y | **Y**  Y | **Y**  Y | **Y** Y | **Y** Y |
| Are the research questions suited to qualitative inquiry? | **1** 2 | **Y**  Y | **Y**  Y | **Y**  Y | **Y**  Y | **Y**  Y | **Y**  Y | **Y**  Y | **Y**  Y | **Y** Y | **Y** Y |
| Is the sampling clearly described? | **1** 2 | **Y**  Y | **N**  Y | **Y**  Y | **Y**  Y | **N**  N | **Y**  Y | **Y**  Y | **Y**  Y | **Y** Y | **Y** Y |
| Is the sampling appropriate to the research question? | **1** 2 | **Y**  Y | **Y**  Y | **Y**  Y | **Y**  Y | **-**  - | **Y**  Y | **Y**  Y | **Y**  Y | **Y** Y | **Y** Y |
| Is the data collection clearly described? | **1** 2 | **Y**  Y | **Y**  Y | **Y**  Y | **Y**  Y | **Y**  Y | **Y**  Y | **Y**  Y | **Y**  Y | **Y** Y | **Y** Y |
| Is the data collection appropriate to the research question? | **1** 2 | **Y**  Y | **Y**  Y | **Y**  Y | **Y**  Y | **Y**  Y | **Y**  Y | **Y**  Y | **Y**  Y | **Y** Y | **Y** - |
| Is the analysis clearly described? | **1** 2 | **Y**  Y | **N**  Y | **Y**  Y | **Y**  Y | **N**  N | **Y**  Y | **Y**  Y | **Y**  Y | **Y** Y | **Y** Y |
| Is the analysis appropriate to the research question? | **1** 2 | **Y**  Y | **Y**  Y | **Y**  Y | **Y**  Y | **Y**  Y | **Y**  Y | **Y**  Y | **Y**  Y | **Y** Y | **Y** Y |
| Are the claims made supported by sufﬁcient evidence? | **1** 2 | **Y**  Y | **Y**  Y | **Y**  Y | **Y**  Y | **Y**  Y | **Y**  Y | **Y**  Y | **Y**  Y | **Y** Y | **Y** Y |
| Are the data, interpretations, and conclusions clearly integrated? | **1** 2 | **Y**  Y | **Y**  Y | **Y**  Y | **Y**  Y | **Y**  Y | **Y**  Y | **Y**  Y | **Y**  Y | **Y** Y | **Y** Y |
| Does the paper make a useful contribution? | **1** 2 | **Y**  Y | **Y**  Y | **Y**  Y | **Y**  Y | **Y**  - | **Y**  Y | **Y**  Y | **Y**  Y | **Y** Y | **Y** Y |
| Overall rating | **1** 2 | **KP**  KP | **SAT**  KP | **KP**  KP | **KP**  KP | **SAT**  SAT | **KP**  KP | **KP**  KP | **KP**  KP | **SAT** SAT | **SAT** UNS |
| Agreed rating |  | **KP** | **SAT** | **KP** | **KP** | **SAT** | **KP** | **KP** | **KP** | **SAT** | **SAT** |

**‡**Criteria met: Yes (Y), No (N), Can’t tell (-)

**§**Quality Rating: **KP:** Key paper - meets all quality criteria and clearly ﬁts with review question. **SAT**: Satisfactory - meets most quality criteria and ﬁts well to review question. **UNS**: Unsatisfactory - unsure mixed responses to quality criteria and lack of clarity regarding relevance to review question

**Appendix 4 (Continued).** *Quality appraisal of included studies 11-20*

| **Prompt** | **Reviewer** | Vandewalle et al  (2019) | Nielsen et al  (2018) | Nyman et al  (2019) | Rimondini et al (2019) | Vandewalle et al  (2019) | Coffey et al  (2019) | Lees et al  (2014) | Hagen et al  (2017) | Fletcher (1999) | Nolan & Quinn  (2012) |
| --- | --- | --- | --- | --- | --- | --- | --- | --- | --- | --- | --- |
| Are the research questions clear? | **1** 2 | **Y** Y | **Y** Y | **Y** Y | **Y** Y | **Y** Y | **Y** Y | **Y** Y | **Y** Y | **Y** Y | **Y** Y |
| Are the research questions suited to qualitative inquiry? | **1** 2 | **Y** Y | **Y** Y | **Y** Y | **Y** Y | **Y** Y | **Y** Y | **Y** Y | **Y** Y | **Y** Y | **Y** Y |
| Is the sampling clearly described? | **1** 2 | **Y** N | **Y** Y | **Y** N | **N** N | **Y** N | **Y** Y | **N** N | **Y** Y | **N** N | **Y** Y |
| Is the sampling appropriate to the research question? | **1** 2 | **Y** - | **Y** Y | **Y** - | **-** - | **Y** Y | **Y** Y | **Y** Y | **Y** Y | **-** - | **Y** Y |
| Is the data collection clearly described? | **1** 2 | **Y** Y | **Y** Y | **Y** Y | **Y** Y | **Y** Y | **Y** Y | **Y** Y | **Y** Y | **N** N | **Y** Y |
| Is the data collection appropriate to the research question? | **1** 2 | **Y** Y | **Y** - | **Y** Y | Y Y | **Y** Y | **Y** Y | **-** N | **Y** Y | **-** - | **Y** Y |
| Is the analysis clearly described? | **1** 2 | **Y** Y | **Y** Y | **Y** Y | Y Y | **Y** Y | **Y** Y | **Y** Y | **Y** Y | **Y** Y | N N |
| Is the analysis appropriate to the research question? | **1** 2 | **Y** Y | **Y** Y | **Y** N | Y Y | **Y** Y | **Y** Y | **Y** - | **Y** Y | **Y** Y | **-** - |
| Are the claims made supported by sufﬁcient evidence? | **1** 2 | **Y** Y | **Y** Y | **Y** Y | Y Y | **Y** Y | **Y** Y | **Y** Y | **Y** Y | **Y** Y | **Y** Y |
| Are the data, interpretations, and conclusions clearly integrated? | **1** 2 | **Y** Y | **Y** Y | **Y** Y | Y Y | **Y** Y | **Y** Y | **Y** Y | **Y** Y | **Y** Y | **Y** Y |
| Does the paper make a useful contribution? | **1** 2 | **Y** Y | **Y** Y | **Y** Y | Y Y | **Y** Y | **Y** - | **Y** Y | **Y** Y | **-** - | **Y** Y |
| **Overall rating** | **1** 2 | **KP** SAT | **SAT** SAT | **SAT** SAT | **SAT** KP | **KP** SAT | **SAT** UNS | **SAT** SAT | **SAT** SAT | **SAT** UNS | **SAT** SAT |
| **Agreed rating** |  | **KP** | **SAT** | **SAT** | **KP** | **KP** | **SAT** | **SAT** | **SAT** | **SAT** | **SAT** |

**‡**Criteria met: Yes (Y), No (N), Can’t tell (-)

**§**Quality Rating: **KP:** Key paper - meets all quality criteria and clearly ﬁts with review question. **SAT**: Satisfactory - meets most quality criteria and ﬁts well to review question. **UNS**: Unsatisfactory - unsure mixed responses to quality criteria and lack of clarity regarding relevance to review question
